# Leveraging Quick Response Codes in Urology to Promote Patient Education While Contributing to Sustainable Healthcare Practices: A Retrospective Study

**DOI:** 10.7759/cureus.71247

**Published:** 2024-10-11

**Authors:** Mohamed S Mohsin, Nader F Gaballa, Banan Osman, Ivo Dukic

**Affiliations:** 1 Department of Urology, University Hospitals Birmingham NHS Foundation Trust, Birmingham, GBR

**Keywords:** lower urinary tract symptoms, patient counseling, patient information leaflet, pre-operative education, quick response (qr) codes, urology consults

## Abstract

Background

Providing patients with sufficient information about the planned procedure they will undergo, including the risks, benefits, and appropriate alternatives, forms a cornerstone of the high standards of care that a urologist provides. Our current standard for delivering information is printed patient information leaflets (PILs) following a consultation with a urologist in a clinic or via telemedicine. Our study considers the feasibility and potential of leveraging quick response (QR) codes for PILs to promote sustainable healthcare practices among urologists.

Methods

The authors conducted a retrospective study, including all patients who attended the one-stop lower urinary tract symptom clinic in a single month in 2024.

Results

In our study, 73.2% of respondents had used a QR code in the past, and 65.9% of patients preferred to use PILs generated with a QR code as their primary source of information regarding their procedure.

Conclusion

QR codes can be effectively promoted among patients, with printed PILs offered to those preferring physical copies. With the increasing uptake and use of smartphones, QR code-generated PILs have the potential to enhance patient education and improve sustainability in healthcare.

## Introduction

Quick response (QR) codes were developed by Denso Wave (DENSO Corporation, Kariya, Japan) for the automotive industry in 1994 [1]. QR codes can be considered “fingerprints” or unique generated codes readable only by software to direct the end user to the primary source of information. QR codes are also superior to standard barcodes, as integrating a vertical component in the former allows up to 350 times more data to be coded onto a QR code [2]. At present, QR codes can be read by the majority of smartphones via the default camera app and were integrated into the camera app by Apple Inc. (Apple, Inc., Cupertino, CA, USA) as early as 2017 [3].

Urologists provide highly specialised care to a diverse group of patients of all ages, and patient counselling provides relevant information on surgical procedures, including the benefits and risks of the proposed operation, which educates and promotes patient understanding of the procedure, allowing them to make an informed decision surrounding their management.

The Royal College of Surgeons of England (RCSEng) outlines a standard of practice for surgeons to follow in consenting patients as part of “Good Surgical Practice” [4]. Pre-operative counselling has a positive influence on post-operative pain and stress, as patients will have a better understanding of the procedure they undergo, as well as their proposed recovery [5].

The mainstay of patients receiving information in urology clinics in the United Kingdom is via a consultation with a urologist or clinical nurse specialist, in person, within an outpatient setting, outlining the diagnosis and the proposed surgical procedure. To further improve the patient’s understanding of the operation, a printed patient information leaflet (PIL) is provided for the patient to explore on their return home.

In our centre, the PILs provided to patients are sourced primarily from the British Association of Urological Surgeons (BAUS) and the European Association of Urology (EAU) websites. As of July 4, 2024, there are 199 information leaflets available, belonging to 12 categories on the BAUS website [6], and 47 sections containing resources for patients available on the EAU website [7]. The aim of this study was to understand patients' sentiments and willingness to shift to digital resources from the current standard of physical PILs. The objective of this study was to determine if QR codes can be utilised to disseminate PILs and resources. 

## Materials and methods

We conducted a retrospective study, which included patients who attended the one-stop lower urinary tract symptoms (LUTS) clinic in a single month (March) in 2024. The LUTS clinic was selected because LUTS contributes to a significant workload for a general urologist, with up to 41% of patients attending a urology clinic presenting with LUTS [8].

All patients who attended the clinic were provided with anonymised feedback forms, which have been tabulated in the Appendices (Table 4). The patient contribution was on a voluntary basis, and due to the lack of patient identifiers, which the authors deemed non-essential, the authors were blinded to which patients opted to participate. Furthermore, our study design deemed that an exclusion criterion was not applicable. To avoid inconveniencing patients, the survey was disseminated while they awaited their appointment.

To keep costs to a minimum and our survey as sustainable as possible, two feedback forms were printed on a single recycled A4 paper sheet. A tablet computer was intentionally not utilised to allow all participants an equal opportunity to complete the survey and to mitigate potential bias.

Data collected from patients included age, gender, use of a smartphone and the internet, prior use of QR codes to access information, a sample QR code to assess ease of access linked to the BAUS patients' homepage, the patient’s standard modality of receiving information from their specialist, and the patient’s future preferences.

## Results

Of the 50 patients included in our study, 43 (86%) were male. Additionally, 41 (82%) of the 50 patients had access to a smartphone and the internet, compared to nine (18%) who did not. Furthermore, 29 (58%) patients relied on information via printed leaflets provided by the Department of Urology, while 21 (42%) patients opted for self-directed internet searching as their primary source of information (Table 1).

**Table 1:** Demographics, smartphone access, standard modality of receiving information about procedures and their preference to adopt QR codes in the future. PIL: patient information leaflet; QR: quick response

Parameters assessed	Responses (n = 50)	
Age (years)	65.5 (17~91)	
Gender	Males	43 (86%)
	Females	7 (14%)
Access to a smartphone	Yes	41 (82%)
	No	9 (18%)
Standard modality of receiving information	PIL	29 (58%)
	Internet	21 (42%)
Preference to adopt QR codes in the future	Yes	29 (58%)
	No	21 (42%)

Of note, amongst the nine non-smartphone users, all nine (100%) patients were older than 70 years of age. Additionally, 13 (87%) of the patients who were 60 years old and younger preferred QR codes as a primary means of receiving information.

As a sub-group analysis of 41 patients in the cohort of smartphone users, 30 (73.2%) patients had used a QR code in the past, 38 (92.7%) were able to access the sample QR code provided with no assistance, and 27 (65.9%) of smartphone users preferred to adopt QR code-directed information as their primary source of receiving information. In comparison, 29 (58%) of all participants included in the study preferred QR codes from an information delivery perspective (Table 2).

**Table 2:** Subgroup analysis amongst smartphone users. QR: quick response

Parameters assessed	Responses (n = 41)	
Prior use of a QR code	Yes	30 (73.2%)
	No	11 (26.8%)
Ability to access the QR code	Yes	38 (92.7%)
	No	3 (7.3%)
Preference to adopt QR codes	Yes	27 (65.9%)
	No	14 (34.1%)

## Discussion

**Figure 1:**
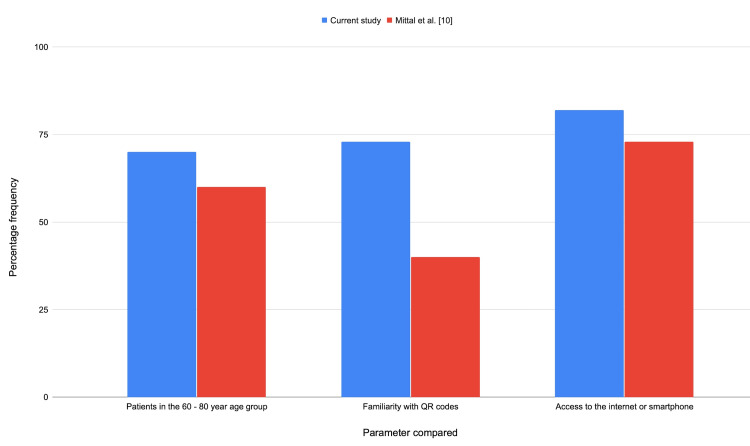
Column chart comparing key findings in the current study versus a study from 2022. QR: quick response Image source: Mittal et al. (2022) [10]

Over the past decade, and certainly since the COVID-19 pandemic [3], where “touch-free” or “contactless” access to information was pivotal in curbing the spread of the virus, the use of QR codes has become more widespread and is viewed as a means of accessing information easily, safely, and sustainably. The benefits of using QR codes are numerous, including convenience and accessibility, medico-legal significance, cost reduction [9], and, importantly, as is the focus of this study - sustainability. Compared to a previous study [10], which analysed the use of QR codes in urology outpatient departments, our study included more patients in the 60 to 80-year age group and demonstrated that not only are more patients now familiar with QR codes, but also that more patients have access to the internet or a smartphone (Figure 1; Table 3).

**Table 3:** Comparison of our results to a study from 2022 QR: quick response Table source: Mittal et al. (2022) [10]

Parameter compared	Result (expressed as a percentage of all patients)	
Patients in the 60-80 year age group	Current study	70%
	Mittal et al. (2022)	60%
Familiarity with QR codes	Current study	73%
	Mittal et al. (2022)	40%
Access to the internet or smartphone	Current study	82%
	Mittal et al. (2022)	73%

**Figure 2:**
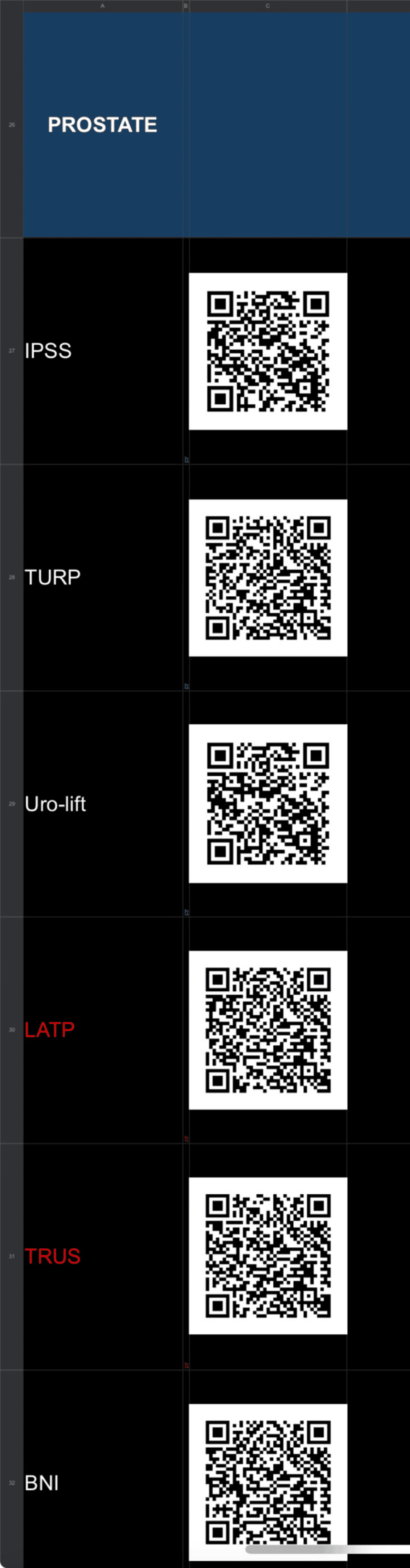
Sample of the author's spreadsheet for PILs related to the prostate PILs: patient information leaflets; IPSS: International prostate symptom score; TURP: transurethral resection of the prostate; Uro-lift: UroLift system; LATP: laparoscopic-assisted transperitoneal prostatectomy; TRUS: transrectal ultrasound; BNI: bladder neck incision

At the authors’ centre, we have successfully generated and used a spreadsheet with QR codes directing the user to the intended, relevant BAUS leaflet and educational videos for the appropriate procedures (Figure 2). The spreadsheet is particularly useful for emergency admissions, where information and videos can be easily shared with patients by simply scanning the QR code on the doctor’s phone. The QR codes were generated on Google Sheets (the Google Suite spreadsheet; Google, Inc., Mountain View, CA) using a simple formula that does not require any existing knowledge of coding. Google Sheets was chosen because the spreadsheet can be accessed on a mobile device or computer at any time, stored offline, and converted to a portable document format (PDF) file if the doctor prefers. Furthermore, in hospitals where the department has developed internal information leaflets that are specific to the region and services provided, a QR code can be generated to link the patient to the relevant local resource.

In a previous study [9], we found that, for a typical LUTS clinic in our centre led by BO, an average of 12 PILs for transurethral resection of the prostate (TURP) are provided per month. When extrapolated, this equates to 144 leaflets per annum or 1,008 pages. 

Through calculations elaborated on in our previous work, we found that the emissions of carbon dioxide (CO2) for a single PIL on TURP, consisting of seven pages, equalled 27 grams, which might seem insignificant at first. However, when extrapolated over a year, emissions amount to 3,900 grams of CO2 for a single type of leaflet. Furthermore, the CO2 emissions generated from this single PIL increase when factors such as postage are taken into account. We propose that the CO2 emissions detailed above can be minimised through the digitisation of PILs - namely, through the adaptation of QR codes.

Limitations of our study

Our study consisted of 50 patients, with a significant proportion being men. Furthermore, our data is from a single centre in the United Kingdom. We recognise that feedback from the female population of patients is underpowered in this particular patient group. Therefore, we suggest larger, multi-centre studies to account for regional variations in patient demographics and preferences, with more female patients included. 

## Conclusions

Notwithstanding the small number of patients included in our study, our results confirm an increase in familiarity with QR codes and access to the Internet, compared to a previous study conducted in 2022.

Our study proves that patients being seen for LUTS are willing to accept digital resources and access them with smartphone QR codes. The authors encourage urologists to offer all patients the option of scanning a QR code to avoid discrimination based on age and other factors, and to actively promote the use of QR codes amongst all patients with access to a data-enabled smartphone, thereby enhancing patient education while contributing to sustainable healthcare practices. The authors identify that, at present, printed PILs may remain the mainstay of delivering information to elderly patients. However, the uptake of digitalised information in the ageing population will likely mirror the increase in smartphone use in the decades to come.

## Appendices

The survey used in this study has been provided below in Table 4. A detailed description of the survey has been provided as a part of the methodology.

**Table 4:** Survey disseminated to patients QR: quick response

Parameter assessed	Data collected		
Date	_____/______/ 2024		
Age	_____________Years		
Gender	_____________________________________________		
Please use your phone camera to scan the code	Sample QR code provided linked to the British Association of Urological Surgeons (BAUS) Website		
Do you use a smartphone?	YES	NO	
Have you used a quick response (QR) code before?	YES	NO	
Were you able to access the website through the code above?	YES	NO	
Would you prefer to receive information leaflets through QR codes instead of printed leaflets?	YES	NO	
How do you usually receive information?	VERBAL	LEAFLETS	INTERNET SEARCH
